# Depression and Fitness: The Role of Psychopharmacology

**DOI:** 10.1192/j.eurpsy.2025.2068

**Published:** 2025-08-26

**Authors:** D. V. Cotovio, R. S. Nogueira, R. Lousada, M. B. Resende, F. Agostinho, M. M. Melo, M. J. Heitor

**Affiliations:** 1 Departamento de Psiquiatria e Saúde Mental, Hospital Beatriz Ângelo, Loures, Portugal

## Abstract

**Introduction:**

The use of antidepressants is becoming more prevalent among athletes due to the growing awareness of mental health issues in sports. However, the impact of these medications, especially selective serotonin reuptake inhibitors (SSRIs), on physical performance remains uncertain. Studies on psychotropic drugs’ effects on athletic capabilities raises concerns about their use in sports, particularly under anti-doping regulations.

**Objectives:**

This review aims to assess the impact of antidepressants on physical exercise performance and muscle metabolism, in order to clarify how they influence physical capabilities.

**Methods:**

A literature search was conducted on PubMed in September 2024 using search terms such as “sports” AND “antidepressants,” “physical activity” AND “antidepressants,” “exercise” AND “selective serotonin reuptake inhibitors,” among others. Only systematic reviews and meta-analyses were included, without restrictions on language or year. Three articles met the scope of this work.

**Results:**

The effects of antidepressants on athletes are inconsistent, with some studies indicating no significant change in performance, while others report reduced endurance. Paroxetine and fluoxetine, commonly prescribed SSRIs, may impair endurance due to increased serotonin levels, which can exacerbate fatigue, known as central fatigue hypothesis. It is also emphasized that SSRIs may reduce athletic performance, especially under thermal stress, by affecting thermoregulation, alongside its interference in serotonin pathways. Potential metabolic impact of these drugs was found, as chronic exposure to SSRIs showed modulation of glucose uptake, mitochondrial respiration, and muscle mass. Furthermore, SSRIs also induced changes in electrical muscle activity.

**Conclusions:**

The evidence on the effects of antidepressants, particularly SSRIs, on physical performance and muscle function remains inconclusive. Athletes and healthcare providers must weigh these risks carefully, considering both the clinical and ethical implications of psychotropic drug use in competitive sports. Therefore, future research should focus on more consistent study protocols and explore the long-term metabolic consequences of SSRIs in physically active populations.

**Disclosure of Interest:**

None Declared

